# Physical activity interventions for cardiopulmonary fitness in obese children and adolescents: a systematic review and meta-analysis

**DOI:** 10.1186/s12887-023-04381-8

**Published:** 2023-11-06

**Authors:** Chaochao Wang, Zuguo Tian, Yuting Hu, Qiaoyou Luo

**Affiliations:** Department of Physical Education, Hunan University, Changsha, Hunan Province 410000 China

**Keywords:** Physical activity, Obesity, Children, Adolescents, Cardiorespiratory fitness

## Abstract

**Purpose:**

This study [PROSPERO CRD42023416272] systematically analysed the effects of a physical activity intervention on cardiorespiratory fitness in obese children and adolescents and elucidated the factors that influenced those effects.

**Methods:**

A systematic review of the literature on physical activity interventions for improving cardiopulmonary fitness in obese children and adolescents from January 1, 2011, to March 1, 2023, was conducted. The search was performed on the Web of Science and PubMed databases, and the selected literature was first screened and then assessed for quality. Finally, a systematic review was conducted.

**Results:**

Out of the initially identified 1424 search records, 28 studies were eventually included in the systematic review. These studies encompassed a total of 2724 participants aged 5 to 18 years, with the publication dates of the literature primarily ranging from 2011 to 2023. Physical activity was found to effectively improve the following parameters in obese children and adolescents: weight [mean difference (MD), -2.03 (95% confidence interval, -2.59 to -1.47), *p* < 0.00001], maximal oxygen consumption [MD, -1.95 (95% CI, -1.06 to -2.84), *p* < 0.0001], heart rate [MD, -2.77 (95% CI, -4.88 to -0.67), *p* = 0.010], systolic blood pressure [MD, -8.11 (95% CI, -11.41 to -4.81), *p* < 0.00001], and diastolic blood pressure [MD, -4.18 (95% CI, -5.32 to -3.03), *p* < 0.00001]. High-intensity exercise was found to yield greater improvements than low- to moderate-intensity exercise in maximal oxygen consumption [MD, 1.43 (95% CI, 0.04 to 2.82), *p* = 0.04] and diastolic blood pressure [MD, -6.94 (95% CI, -10.61 to -3.26), *p* = 0.0002] in obese children and adolescents.

**Conclusion:**

Physical activity can effectively improve the body weight, maximal oxygen consumption, heart rate, systolic blood pressure, and diastolic blood pressure of obese children and adolescents. The type of physical activity directly influences the participation interest of obese children and adolescents, with moderate- to high-intensity physical activity showing the most significant impact on intervention outcomes. High-frequency, long-term interventions yield better results than short-term interventions.

**Supplementary Information:**

The online version contains supplementary material available at 10.1186/s12887-023-04381-8.

Cardiorespiratory fitness (CRF) is one of the most critical aspects of physical fitness [1], and is primarily defined characterized by maximum oxygen uptake (VO_2max_) or metabolic equivalents (METs) [2–4]. Lower levels of CRF are associated with cardiovascular disease (CVD) and cancer, as well as higher morbidity and mortality [5]. Childhood CRF affects overall health status during youth and can also reduce the risk of cardiovascular disease [6]. Higher levels of CRF in children and adolescents are associated with a healthier cardiovascular status [7]. Childhood obesity is a global health problem, with the prevalence of obesity increasing every year and obesity inducing many other conditions [8]. Overweight and obese children have lower CRF levels, which increases the risk of CVD in adulthood [9]. CRF in obese children and adolescents is closely linked to physical activity, and physical activity can significantly alter CRF levels in this population [10–13]. Therefore, an increasing number of studies have begun to explore effective ways to improve cardiorespiratory health in obese children and adolescents.

Physical activity (PA) is defined as “any physical movement of skeletal muscle that results in energy expenditure” [14]. When the body is physically active, the respiratory and circulatory systems provide the body with energy and transport metabolic substances. Lack of PA, poor diet and other factors such as a sedentary lifestyle are associated with an alarming increase in overweight and obesity in children. Therefore, the World Health Organisation recommends that children accumulate 60 min of moderate- to high -intensity PA per day [15]. In terms of interventions, some scholars have noted that an increase in school physical activity has beneficial effects on CRF in obese children and adolescents [16]. Many experimental studies comparing the effects of high-intensity interval training (HIIT) and moderate-intensity continuous training (MICT) on CRF in children and adolescents have shown significant benefits of HIIT interventions [17, 18]. To date, there is no literature that reviews and elucidates the factors influencing CRF in obese children and adolescents and the effect of PA on improving CRF in obese child adolescents.

Therefore, the aim of this study was to review the literature on CRF affecting adolescents with obesity and to identify the effects of PA interventions while analysing potential moderators. The results of this study provide theoretical references and recommendations for future intervention strategies for CRF in obese children and adolescents.

## Materials and methods

This systematic review has been registered with Prospero, the International Prospective Register of Systematic Reviews (Registration number: CRD42023416272).

### Literature search and data extraction

We conducted a systematic literature search using PubMed and Web of Science for studies up to March 2023 that investigated the impact of exercise interventions on CRF in adolescents with obesity and were published in English. We searched the database using the following search terms, and key terms, including Medical Subject Headings (MeSH) terms. MeSH terms used included ‘physical activity (PA)’, ‘exercise’, ‘physical activity’, ‘obesity’, ‘overweight’, ‘children’, ‘adolescents’ and ‘cardiorespiratory fitness’. The detailed search strategy is presented in Supplementary Table (see Additional file 1).

### Literature inclusion and exclusion criteria

This systematic review was guided by the PRISMA checklist [19]. The first step was to identify the core concepts in the research question, followed by a systematic search. The study population was required to meet the following criteria: School-aged children and adolescents (5–18 years) who were obese or overweight were eligible for the study. Regardless of the age range of the study sample, the mean participant age of an eligible study must have been in the 5–18 years age range. For example, a study sample was included if the participants had an age range of 11–20 years and a mean age equal to 15 years. The study intervention was required to meet the following criteria: PA, physical education classes or promotion of PA to increase the behaviour of PA. The study conclusion was required to meet the following criteria: exercise-based CRF measurement with appropriate analysis of the effect on CRF (i.e. pretest to posttest compared to the control group). The study design was required to be either a randomised controlled trial (RCT) or a quasi-experimental design (QES). The study exclusion criteria were as follows: the intervention was not restricted to children with obesity; and qualitative studies, case studies, reviews, nonintervention studies, master's theses, conference papers.

### Literature screening and data extraction

The retrieved literature was imported into EndNote software for deduplication and then 2 researchers (CCW and QYL) independently read the title, abstract and full text for literature screening. When disagreements arose, the final results were determined by consensus with a third researcher (ZGT). Based on the literature screening, the two researchers proceeded to extract and code the literature information including author, country, year of publication, study population, intervention content, intervention protocol (timing, frequency and periodicity), measurement tools and outcome indicators.

### Evaluation of the quality of the literature

The Physiotherapy Evidence Database (PEDro) scale was used to assess the methodological quality of the included literature. Eleven items were included, including “eligibility criteria”, “random allocation”, “concealed allocation”, “baseline comparability”, “blind subject”, “blind clinician”, “blind assessor”, “adequate follow-up”, “intention-to-treat analysis”, “between-group analysis”, and “point estimates and variability”. Items 2 to 11 are scored, with 1 mark for meeting the criteria and 0 mark for not meeting or being unclear. The scale was scored out of 10, with < 4 being poor quality, 4–5 being moderate quality, 6–8 being good quality and 9–10 being high quality. The quality of the literature was scored by two researchers independently, and in case of disagreement, the final decision was made by a third researcher in a joint discussion.

### Data synthesis and analysis

Evidence synthesis was conducted using Review Manager 5.3 (Cochrane Collaboration, Oxford, UK). Weight, maximal oxygen consumption, heart rate, systolic blood pressure, and diastolic blood pressure were analysed as continuous variables. We selected the mean differences (MDs) and 95% confidence intervals (CIs) as summary statistics for the meta-analysis. Heterogeneity among studies was assessed using the chi-square (Χ^2^) test (Cochran's Q) and the inconsistency index (I^2^) [20]. A Χ^2^ *p* value < 0.05 or I^2^ > 50% was considered to indicate significant heterogeneity. In the presence of significant heterogeneity, a random-effects model was employed. Otherwise, a fixed-effects model was applied. A funnel plot was created using Review Manager 5.3 (Cochrane Collaboration, Oxford, UK). Outcomes were assessed in at least two included RCTs.

## Research results

**Fig. 1:**
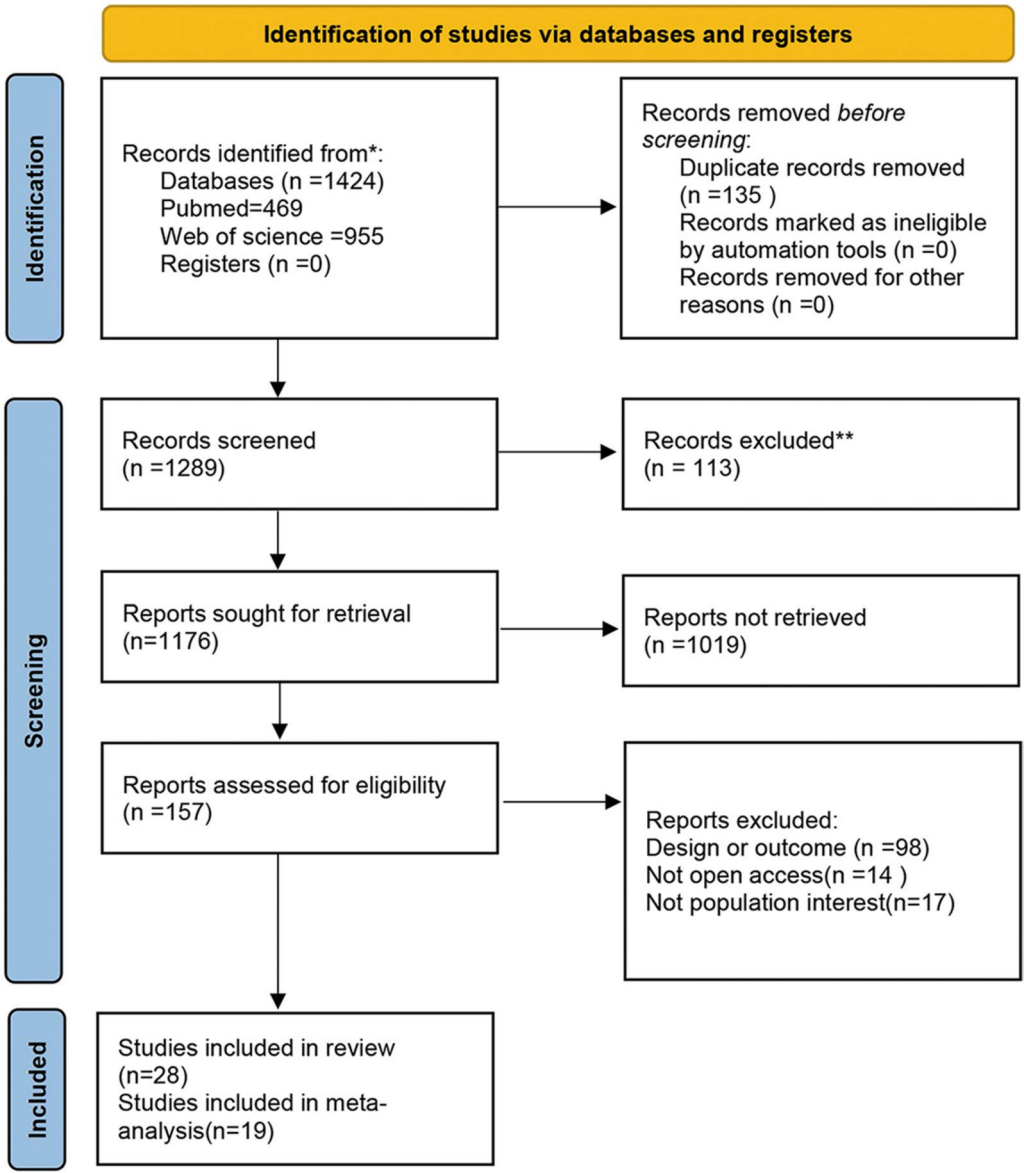
Flowchart detailing the systematic search, screening, eligibility, and inclusion procedure

A total of 1424 studies were identified by the search. After excluding 135 duplicate studies, 1289 documents were screened by title and abstract. After 1132 studies were disqualified for reasons such as noncompliance with the article topic and study design, the remaining 157 studies were reviewed in full. After a review and thorough reading of the remaining studies, 28 papers were ultimately include [10–13, 16, 18, 21–42] (Fig. 1).

### Basic characteristics of included studies

This review included 28 studies (Table 1): 8 studies were conducted in Europe (1 from Italy, 1 from Serbia, 1 from the Netherlands, 1 from Denmark, 2from France, 1 from Norway, and 2 from Spain), 6 studies in South America (1 from Chile, 5 from Brazil), 5 studies in North America (4 from the United States, 1 from Canada), 6 studies in Asia (2 from China, 1 from Iran, 3 from South Korea), 2 studies in Africa (all from Tunisia), and 1 study in Oceania (from Australia). The publication dates primarily ranged from 2011 to 2023. The earliest publication among the included studies was in 2011 [21], and the most recent study was published in 2023 [42]. The study designs mainly consisted of 25 randomised controlled trials (RCTs), 2 quasi-experimental studies (QES), and 1 nonrandomised controlled trial (non-RCT). The total number of participants was 2724, all of whom were obese children and adolescents. The largest number of participants in a single study was 574 [23], while the smallest was 13 [30, 42]. In almost all of the studies, participants included both males and females. However, in 5 studies [12, 29, 31, 36, 42], participants were exclusively male. In 3 studies [25, 33, 34], all participants were female.

**Table 1:** Basic characteristics of the included literature

Study Reference -Design-	Country	Number of Subjects	Age (range or mean range)	Intervention Description	Intensity	Intervention Duration	Measurement program	Duration of Intervention (week)	Results
		Sex							
Thivel et al.(2011) [21] RCT	France	N:101	6–10	E:PE + AL C:PE	N/A	60 min 2times weekly	Shuttle run test cycle peak power	24	Wight*, HRR*, RHR* (E&C)
Coute de Araujo et al. (2012) [22] RCT	Brazil	N:30	8–12 岁	Endurance exercise: walking/running exercise HIIT: Treadmill sprints	Endurance exercise: Medium intensity HIIT: high intensity	twice a week	A modified Balke treadmill	12	Wight*, SBP*, DBP* (HIIT)
Yin et al. (2012) [23] RCT	USA	N:574	8.7 ± 0.5	Daily, Kids4Fit	Medium to high intensity	120 min daily	Step test	156	HR* (E&C)
Koubaa et al.(2013) [24] RCT	Tunis	N:29	13 ± 0.8	HIIT, CT (Continuous training)	IT:80% of the VO_2max_ CT:60% to 70% of VO_2max_	3 times weekly	dynamic test	12	Wight*, SBP*(E)
Racil et al. (2013) [25]	Tunis	N:34 F:34	15.9 ± 0.3	HIIT: running MIIT: running C: No intervention	Medium or high intensity	3 times weekly	The maximal test	12	VO_2max_*
Khan et al.(2014) [26] RCT	USA	N:220 M:117 F:103	8–9	E: MVPA C: No intervention	Medium to high intensity	70 min 5 times weekly	Treadmill protocol	39	Wight*, VO_2max_* (E)
Farah et al. (2014) [27] RCT	Brazil	N:19 M:9 F:10	15.1 ± 1.2	HIT, LIT (Aerobic training on a treadmill)	HIT:100%VT LIT:80% VT	3 times weekly	Treadmill protocol	24	Wight*, HR*, HRV* (HIT) SBP*, DBP* BP* (HIT&LIT)
Alberga et al. (2015) [28] RCT	Canada	N:151	14–18	AE C: No intervention	medium intensity	N/A	the 2003 Canadian Physical Activity Fitness and Lifestyle Appraisal tests	22	Wight*, VO_2max_* (A)
Tan et al. (2015) [29] RCT	China	M:24	8–10	E: Daily PA C: No intervention	individualized FATmax intensity	40 min 5 times weekly	Shuttle run test	10	Wight*, VO_2max_* (E)
Murphy et al. (2015) [30] RCT	USA	N:13 M:3 F:10	13.7 ± 2.0	AE HIIT	65% HRmax 80–90%HRmax	50 min 3 times weekly	Motorized Treadmill Protocol	4	VO_2max_* (HIIT & AE)
Martinez et al. (2016) [16] RCT	Spain	N:94 M:52 F:42	7–9	E:PE + AL C:PE	High intensity	90 min 2 times weekly	Shuttle run test Treadmill protocol	13	VO_2max_* EPOC* (E)
Lazzer et al. (2016) [31] RCT	Italy	N:30 M:30	13.7 ± 2.0	LA (Walking)	HIIT:100%VO_2max_ High intensity:70%VO_2max_ LIT:40%VO_2max_	37 ± 3 min 31 ± 4 min 45 ± 6 min 3 times weekly	Motorized Treadmill Protocol	3	Wight*, VO_2peak_* (HIIT & High intensity)
Kargarfard et al. (2016) [32] RCT	Iran	N:30	12.3 ± 1.3	HIIT ET (Running)	60–90%HRR 60–70%HRR	50-60 min 3 times weekly	Incremental two-phase cycle	8	SBP*, DBP* (HIIT&ET)
Dias et al. (2017) [18] RCT	Australia	N:41 M:21 F:20	12.2 ± 2.1	Walking	HIIT:85–95%HRmx MICT:60–70%HRmax	3 times weekly	Treadmill protocol ramp Respiratory gas analysis	12	Wight*, VO_2peak_* (HIIT)
Son et al. (2017) [33] RCT	Korea	N:40 F:40	15 ± 1	E: Combined exercise C: No intervention	60–70% HRR	60 min- utes per day, 3 times per week	Automatic sphygmomanometer	12	Wight*, SBP*, DBP* (E)
Sung et al. (2018) [34] RCT	Korea	N:40 F:40	14–16	E: Jump rope C: No intervention	medium intensity	50 min per day, 5 times per week	Automatic sphygmomanometer	12	Wight*, SBP*, DBP*, HR* (E)
Ingul et al. (2018) [35] RCT	Norway	N:41 M:21 F:20	12.0 ± 2.3	Cycling	HIIT:85–95%HRmax MICT:60–70%HRmax	3 times weekly	Treadmill Protocol ramp Respiratory gas analysis	12	SBP*, DBP* (HIIT&MICT)
Cvetkovic et al. (2018) [36] RCT	Serbia	N:21 M:21	11–13	The football training HIIT: Running	100% MAS N/R	60 min 3 times weekly	Yo − Yo test distance	12	Wight*, RHR*, DBP*, SBP* (HIIT) DBP*, SBP* (The football training)
Morrissey et al. (2018) [37] non-RCT	France	N:29 M:8 F:21	15.2 ± 1.4	HIIT MICT	90–95% HRmax 60–70% HRmax	24-32 min 40-60 min 3 times weekly	20mShuttle run test bouts	12	Wight*, RHR* (HIIT&MICT) DBP* (MICT)
Leeuwen et al. (2018) [10] QES	Netherlands	N:154 M:66 F:88	8.5 ± 1.8	Kds4Fit	N/A	60 min 2times weekly;(First 6 weeks) 1 times weekly (Last 6 weeks)	Shuttle run test	13	VO_2max_* BP* (E)
Ye et al. (2019) [38] QES	China	N:81 M:42 F:39	9.23 ± 0.62	E:PE + EXG C:PE	N/A	50 min 1/week	Half-mile run	35	/
Davis et al. (2019) [11] RCT	USA	N:75 M:29 F:46	9.5–9.8	E: ASAE C: No intervention	AHR > 140times/min	Daily 40 min	Treadmill protocol Carotid-femoral pulse wave velocity	35	VO_2peak_* (E)
Espinoza-Silva et al. [39] (2019) RCT	Chile	N:274 M:120 F:154	7–9	E: HIIT C: No intervention	High intensity	40–50 min 2x/week	6 min walk test	30	VO_2max_* (E)
Roh et al. (2020) [40] RCT	Korea	N:20	12.55 ± 0.51	E: Taekwondo training C: No intervention	Medium to high intensity	60 min five times a week	colorimetric assay	16	Wight*, VO_2max_* (E)
Leandro et al. (2021) [12] RCT	Brazil	M:41	7–9	E: PLT C: No intervention	Medium to high intensity	20 min 3times weekly	automatic arterial blood pressure monitor	13	BP*, RHR* (E)
Martinez-Viscaiano et al. (2022) [13] RCT	Spain	N:487 M:233 F:254	9.89 ± 0.71	The MOVI-daFit	High intensity	60 min 4times weekly	Shuttle run test	39	BP*, VO_2max_* (E)
Machado et al. (2022) [41] RCT	Brazil	N:56	10.3 ± 1.8	PE + swimming PE + sedentary	Medium to high intensity	50 min 2times weekly	20 m Shuttles run test	48	SBP*, DBP* (Swimming)
Pinho et al. (2023) [42] QES	Brazil	M:13	8–12	RMST	> 80%HRmax	80 min 2times weekly	McMaster All-Out Progressive Continuous Cycling Test	12	/

*N* number of respondents, *M *male participants, *F* female participants, *E* experimental group, *C* Control group, *PA* physical activity, *PE* Physical Education, *Kids4Fit *multidisciplinary weight reduction program, *FT* Football training, *MVPA* moderate-to-vigorous intensity physical activity, *TCT* Treadmill cardio training, *AE* aerobic exercise, *LA* leisure activities, *ET* Endurance training, *EXG* exergaming, *ASAE* after school aerobic exercise, *PLT* plyometric training, *RMST* Recreational Mini Soccer Training Program, *HIIT* high intensity interval training, *MICT* Moderate intensity continuous training, *LIT* Low-intensity training, *VT* Ventilation threshold, *HRmax* maximum heart rate, *VO*_*2max*_ maximal oxygen consumption, *VO*_*2peak*_ peak oxygen uptake, *HRR* Heart rate reserve, *MAS* Maximum aerobic speed, *AHR* Average heart rate, *RHR* rest heart rate, *HR* Heart rate, *HRV* Heart rate variability, *LVSEF* left ventricular systolic ejection fraction, *IVRTglobal* global isovolumetric relaxation time, *BP* blood pressure, *SBP* Systolic blood pressure, *DBP* Diastolic blood pressure, *EPOC* Excess post-exercise oxygen consumption

^*^Significant improvement

### Evaluation of the quality of the included literature

The 28 studies included in the analysis scored 3 to 9 on the PEDro scale, of which 1 scored < 3 [10], 10 scored 4 to 5 [22, 24, 28, 30, 31, 33, 38–40, 42], 15 scored 6 to 8 [11, 12, 16, 21, 23, 25–27, 29, 32, 34–37, 41], and two scored 9 to 10 [13, 18], with an average score of 6.3. The overall quality of the included studies was good (Table 2).

**Table 2:** PEDro scale results

Study	Eligibility criteria	Random allocation	Concealed allocation	Baseline comparability	Blind subject	Blind clinician	Blind assessor	Adequate follow-up	Intention-to-treat analysis	Between-group analysis	Point estimates and variability	Total awarded points
Thivel et al. (2011) [21]	**√**	**√**	**√**	**√**				**√**	**√**	**√**	**√**	7
Coute de Araujo et al. (2012) [22]	**√**	**√**		**√**				**√**		**√**	**√**	5
Yin et al. (2012) [23]	**√**	**√**	**√**	**√**					**√**	**√**	**√**	6
Koubaa et al. (2013) [24]	**√**			**√**		**√**	**√**		**√**	**√**		5
Racil et al. (2013) [25]	**√**	**√**	**√**	**√**	**√**		**√**	**√**		**√**		7
Khan et al. (2014) [26]	**√**	**√**	**√**	**√**	**√**			**√**	**√**	**√**	**√**	8
Farah et al. (2014) [27]	**√**	**√**	**√**	**√**		**√**	**√**	**√**		**√**		7
Alberga et al. (2015) [28]	**√**	**√**	**√**						**√**	**√**	**√**	5
Tan et al. (2015) [29]	**√**	**√**	**√**	**√**				**√**	**√**	**√**	**√**	7
Murphy et al. (2015) [30]	**√**			**√**		**√**				**√**	**√**	4
Martinez et al. (2016) [16]	**√**	**√**	**√**	**√**				**√**	**√**	**√**	**√**	7
Lazzer et al. (2016) [31]	**√**	**√**	**√**	**√**		**√**		**√**				5
Kargarfard et al. (2016) [32]	**√**	**√**	**√**	**√**	**√**	**√**			**√**	**√**	**√**	8
Dias et al. (2017) [18]	**√**	**√**	**√**	**√**	**√**	**√**		**√**	**√**	**√**	**√**	9
Son et al. (2017) [33]	**√**	**√**	**√**							**√**	**√**	4
Sung et al. (2018) [34]	**√**	**√**	**√**		**√**				**√**	**√**	**√**	6
Ingul et al. (2018) [35]	**√**	**√**	**√**	**√**	**√**	**√**		**√**		**√**	**√**	8
Cvetkovic et al. (2018) [36]	**√**	**√**	**√**	**√**	**√**	**√**			**√**	**√**	**√**	8
Morrissey et al. (2018) [37]	**√**		**√**	**√**		**√**		**√**	**√**	**√**	**√**	7
Leeuwen et al. (2018) [10]	**√**							**√**	**√**		**√**	3
Ye et al. (2019) [38]	**√**	**√**	**√**					**√**		**√**	**√**	5
Davis et al. (2019) [11]	**√**	**√**	**√**	**√**	**√**			**√**	**√**	**√**	**√**	8
Espinoza-Silva et al. (2019) [39]	**√**			**√**				**√**	**√**	**√**	**√**	5
Roh et al. (2020) [40]	**√**	**√**						**√**		**√**	**√**	4
Leandro et al. (2021) [12]	**√**	**√**	**√**	**√**				**√**	**√**	**√**	**√**	7
Martinez-Viscaiano et al. (2022) [13]	**√**	**√**	**√**	**√**	**√**	**√**	**√**	**√**		**√**	**√**	9
Machado et al. (2022) [41]	**√**	**√**	**√**	**√**		**√**		**√**	**√**	**√**	**√**	8
Pinho et al. (2023) [42]	**√**			**√**		**√**	**√**	**√**	**√**			5

Items 2 to 11 are scoring items. 1 mark will be given for meeting the standards, and 0 marks will be given for inconformity or unclear

### Choice of physical activity

The choice of activity type, intensity, frequency, and duration significantly impacts the effectiveness of cardiovascular health interventions in obese children and adolescents. In this study, physical activity types encompassed both physical education classes and extracurricular exercises, specifically including moderate-intensity training, high-intensity interval training, strength training, 6-min walk tests, half-mile runs, multidisciplinary weight loss programs, treadmill exercises, sports games, and endurance training. Activity intensity primarily focused on high and moderate intensities, with 17 studies opting for high-intensity activities [13, 16, 18, 22, 24, 25, 27, 30–32, 35–37, 39–42] and 11 studies selecting moderate-intensity activities [16, 18, 22, 24, 25, 28, 33–37, 40]. Nine studies compared high-intensity activities with low- to moderate-intensity activities [18, 22, 24, 25, 27, 31, 35–37]. The duration of single interventions was mainly centred at approximately 60 and 40 min, with six studies employing 60 min [10, 13, 21, 33, 36, 40] and three studies using 40 min [11, 29, 39]. The longest intervention period was 156 weeks [23], while the shortest was 3 weeks [31]. Statistical analysis of the selected literature revealed a diverse range of physical activity types, with walking and running being the predominant choices. From the literature, it was evident that walking and running are conducive to controlling the intensity of physical activity. The majority of physical activity was conducted at a moderate-to-high intensity level, which can have an impact on cardiovascular outcomes.

### Effectiveness of the intervention

A number of programmes are used to derive outcome indicators. The main measurement options are SRT, CPP and TRE. Eight of these use SRT [10, 13, 16, 21, 29, 32, 37, 41], one of which is an incremental two-phase cycle [32]. Nine used TRE [11, 16, 18, 22, 26, 27, 30, 31, 35], of which two used a modified treadmill scheme [30, 31]. Two items used treadmill ramp and respiratory gas analysis [18, 35]. The measurement program was mainly based on running and walking, with a modified program chosen according to the experiment. In terms of outcome indicators, a total of 13 indicators were covered, including Weight, VO_2max_, VO_2peak_, BP, SBP, DBP, HR, HRR, RHR, HRV, LVSEF, IVRTglobal and EPOC. Fourteen studies used Weight as the outcome measure [12, 18, 21, 24, 26–29, 31, 33, 34, 36, 37, 40]. Ten studies used VO_2max_ as the outcome measure [10, 13, 16, 25, 26, 28–30, 39, 40]. Three studies used VO_2peak_ as the outcome measure [11, 18, 31]. Eight studies used SBP and DBP as outcome indicators [22, 24, 32–36, 41]. Five studies used RHR as an outcome indicator [12, 21, 34, 36, 37]. The primary outcome indicators for evaluating the effect of a PA intervention on CRF in obese children and adolescents were VO_2max_, VO_2peak_, SBP, DBP, BP and RHR.

### Results of the meta-analyses

In our review, we primarily conducted meta-analyses for weight, vo_2max_, heart rate, systolic blood pressure, and diastolic blood pressure. Change scores from baseline to final values were used for our final efficacy analysis. The analysis results for each outcome are as follows.

### Weight

**Fig. 2:**
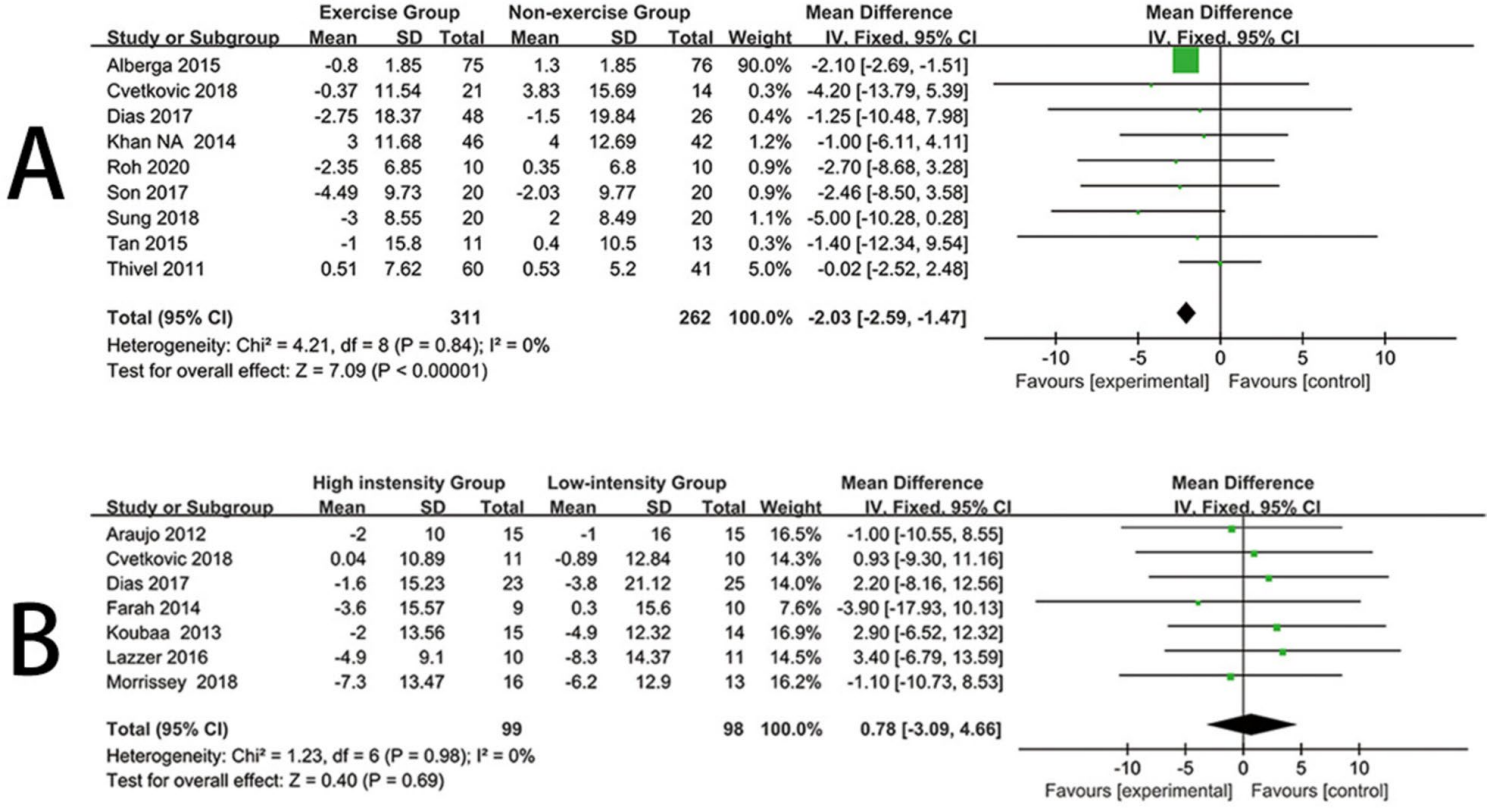
Forest plot for weight. **A** Forest plot of the exercise group vs the nonexercise group. **B** Forest plot of the high-instensity group vs the low-intensity group

Nine trials [18, 21, 26, 28, 29, 33, 34, 36, 40] reported the impact of exercise interventions on weight in obese children and adolescents, comprising 573 participants. Therefore, these nine studies were included in the meta-analysis. Due to low heterogeneity in this review (I^2^ = 0%, *P* = 0.84), a fixed-effects model was used. The results demonstrated strong evidence that exercise interventions significantly lowered the weight levels of obese children and adolescents compared to the control group (mean difference (MD) = -2.03, 95% confidence interval [CI] = [-2.59, -1.47], *P* < 0.00001) (Fig. 2A). Visual interpretation of funnel plots suggested no evidence of asymmetry (see Additional file 1).

Seven trials [18, 22, 24, 27, 31, 36, 37] reported the impact of high-intensity and low-intensity exercise on weight in obese children and adolescents, comprising 197 participants. Therefore, these seven studies were included in the meta-analysis. As there was no heterogeneity in this review (I^2^ = 0%, *P* = 0.98), a fixed-effects model was used. The results indicated that there was no significant difference in weight levels for obese children and adolescents between the high-intensity exercise and low-intensity exercise groups (mean difference (MD) = 0.78, 95% confidence interval [CI] = [-3.09, 4.66], *P* = 0.69) when compared with the low-intensity exercise group (Fig. 2B). Visual interpretation of funnel plots suggested no evidence of asymmetry (See Additional file 1).

### Maximal oxygen consumption

**Fig. 3:**
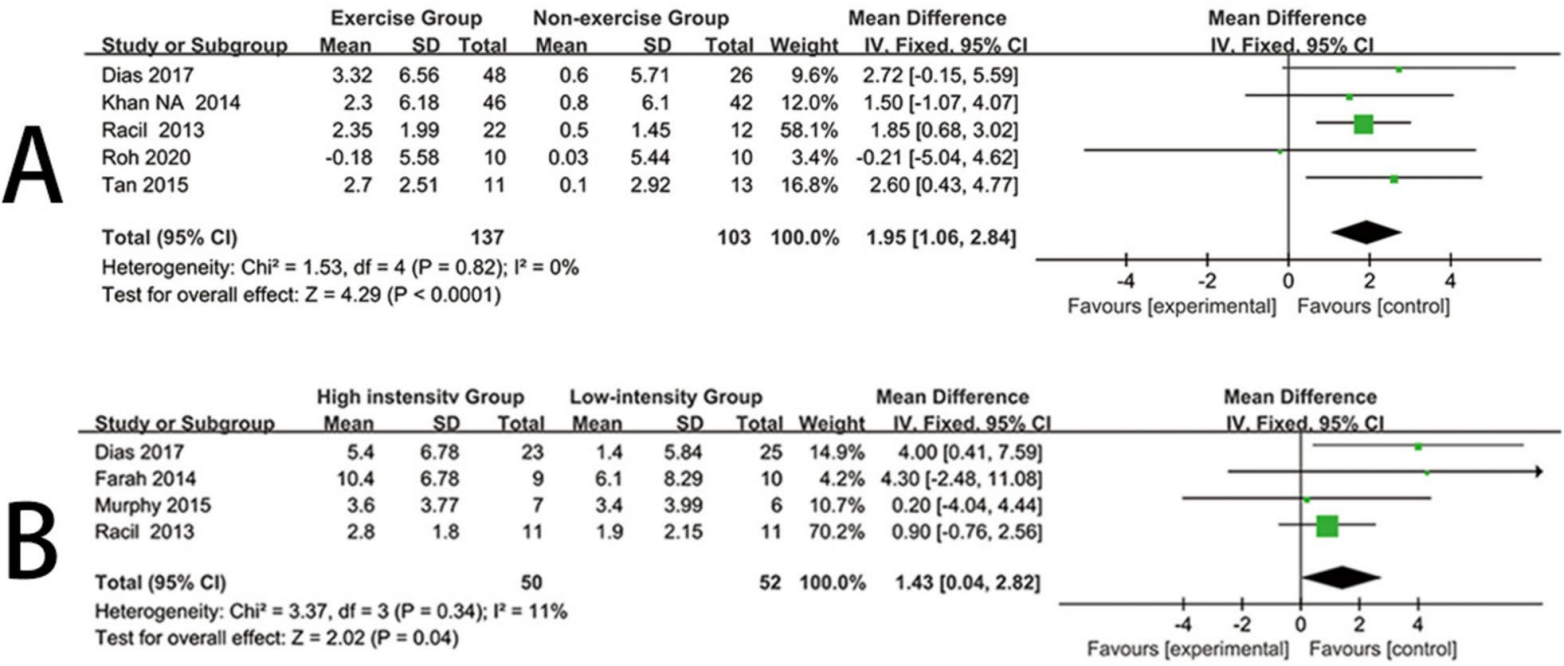
Forest plot for VO_2max_. **A** Forest plot of the exercise group vs the nonexercise group. **B** Forest plot of the high-instensity group vs the low-intensity group

Five trials [18, 25, 26, 29, 40] reported the impact of exercise interventions on VO_2max_ in obese children and adolescents, comprising 240 participants. Therefore, these five studies were included in the meta-analysis. Due to low heterogeneity in this review (I^2^ = 0%, *P* = 0.82), a fixed-effects model was used. The results provided strong evidence that exercise interventions significantly increased the VO_2max_ levels of obese children and adolescents compared to the control group (mean difference (MD) = 1.95, 95% confidence interval [CI] = [1.06, 2.84], *P* < 0.0001) (Fig. 3A). Visual interpretation of funnel plots suggested no evidence of asymmetry (See Additional file 1).

Four trials [18, 25, 27, 30] reported the impact of high-intensity and low-intensity exercise on VO_2max_ in obese children and adolescents, comprising 102 participants. Therefore, these four studies were included in the meta-analysis. Given the low heterogeneity in this review (I^2^ = 11%, *P* = 0.34), a fixed-effects model was used. The results indicated that, compared to low-intensity exercise, high-intensity exercise had a significantly more pronounced effect on improving the VO_2max_ levels of obese children and adolescents (mean difference (MD) = 1.43, 95% confidence interval [CI] = [0.04, 2.82], *P* = 0.04) (Fig. 3B). Visual interpretation of funnel plots suggested no evidence of asymmetry (See Additional file 1).

### Heart rate

**Fig. 4:**
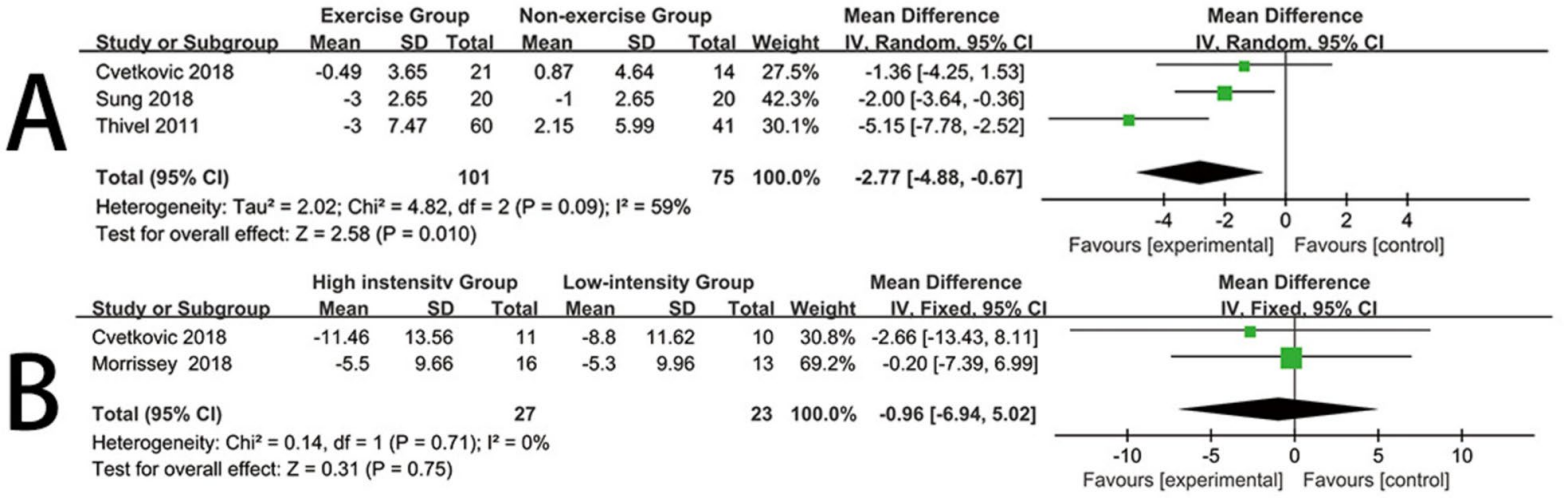
Forest plot for Heart rate. **A** Forest plot of the exercise group vs the nonexercise group. **B** Forest plot of the high-instensity group vs the low-intensity group

Three trials [21, 34, 36] reported the impact of exercise interventions on heart rate in obese children and adolescents, comprising 176 participants. Therefore, these three studies were included in the meta-analysis. Given the moderate heterogeneity in this review (I^2^ = 59%, *P* = 0.09), a random-effects model was used. The results provided strong evidence that exercise interventions significantly lowered the heart rate levels of obese children and adolescents compared to the control group (mean difference (MD) = -2.77, 95% confidence interval [CI] = [-4.88, -0.67], *P* = 0.010) (Fig. 4A). Visual interpretation of funnel plots suggested no evidence of asymmetry (See Additional file 1).

Two trials [36, 37] reported the impact of high-intensity and low-intensity exercise on heart rate in obese children and adolescents, comprising 50 participants. Therefore, these two studies were included in the meta-analysis. As there was no heterogeneity in this review (I^2^ = 0%, *P* = 0.71), a fixed-effects model was used. The results indicated that, compared to low-intensity exercise, high-intensity exercise did not significantly differ in its effect on heart rate levels for obese children and adolescents (mean difference (MD) = -0.96, 95% confidence interval [CI] = [-6.94, 5.02], *P* = 0.75) (Fig. 4B). Visual interpretation of funnel plots suggested no evidence of asymmetry (See Additional file 1).

### Systolic blood pressure

**Fig. 5:**
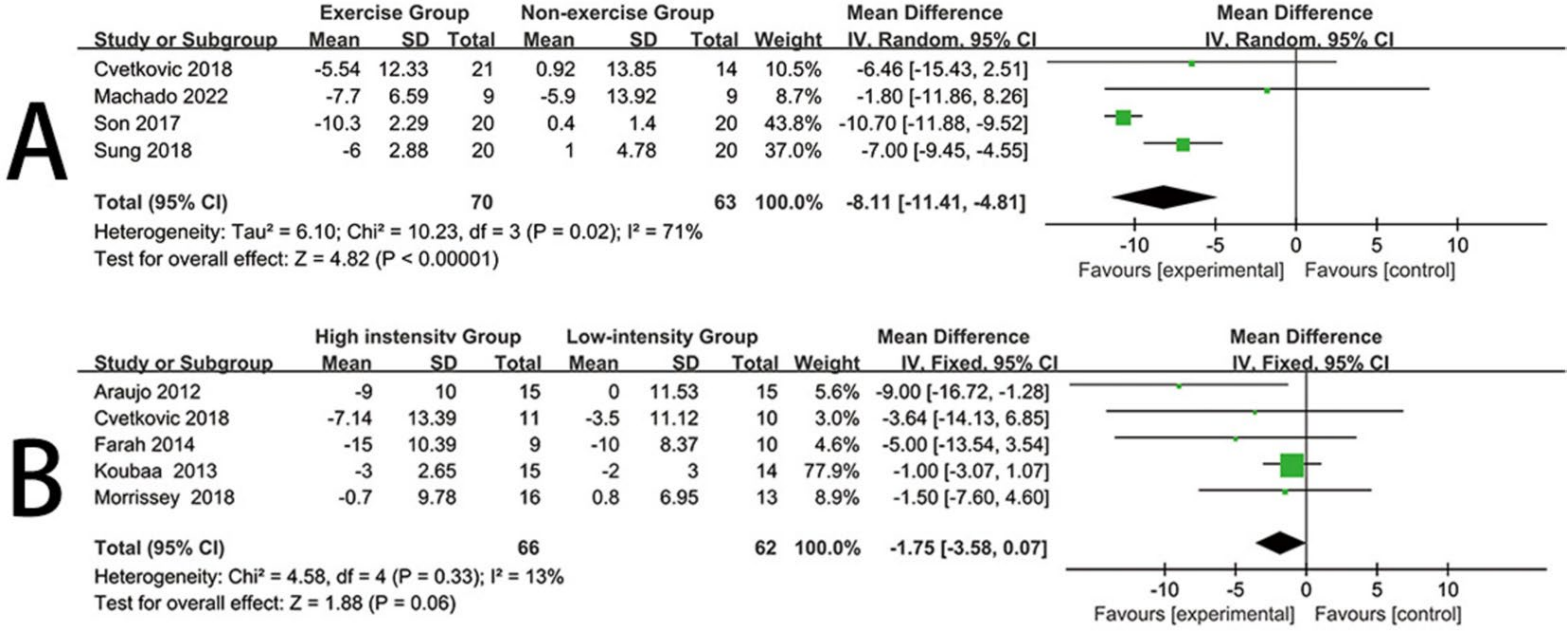
Forest plot for Systolic blood pressure. **A** Forest plot of the exercise group vs the nonexercise group. **B** Forest plot of the high-instensity group vs the low-intensity group

Four trials [33, 34, 36, 41] reported the impact of exercise interventions on systolic blood pressure in obese children and adolescents, comprising 133 participants. Therefore, these four studies were included in the meta-analysis. Given the high heterogeneity in this review (I^2^ = 71%, *P* = 0.02), a random-effects model was used. The results provided strong evidence that exercise interventions significantly reduced the systolic blood pressure levels of obese children and adolescents compared to the control group (mean difference (MD) = -8.11, 95% confidence interval [CI] = [-11.41, -4.81], *P* < 0.00001) (Fig. 5A).

Five trials [22, 24, 27, 36, 37] reported the impact of high-intensity and low-intensity exercise on systolic blood pressure in obese children and adolescents, comprising 128 participants. Therefore, these five studies were included in the meta-analysis. Given the low heterogeneity in this review (I^2^ = 13%, *P* = 0.33), a fixed-effects model was used. The results indicated that, when compared with low-intensity exercise, high-intensity exercise did not result in a significant difference in systolic blood pressure levels for obese children and adolescents (mean difference (MD) = -1.75, 95% confidence interval [CI] = [-3.58, 0.07], *P* = 0.06) (Fig. 5B). Visual interpretation of funnel plots suggested no evidence of asymmetry (See Additional file 1).

### Diastolic blood pressure

**Fig. 6:**
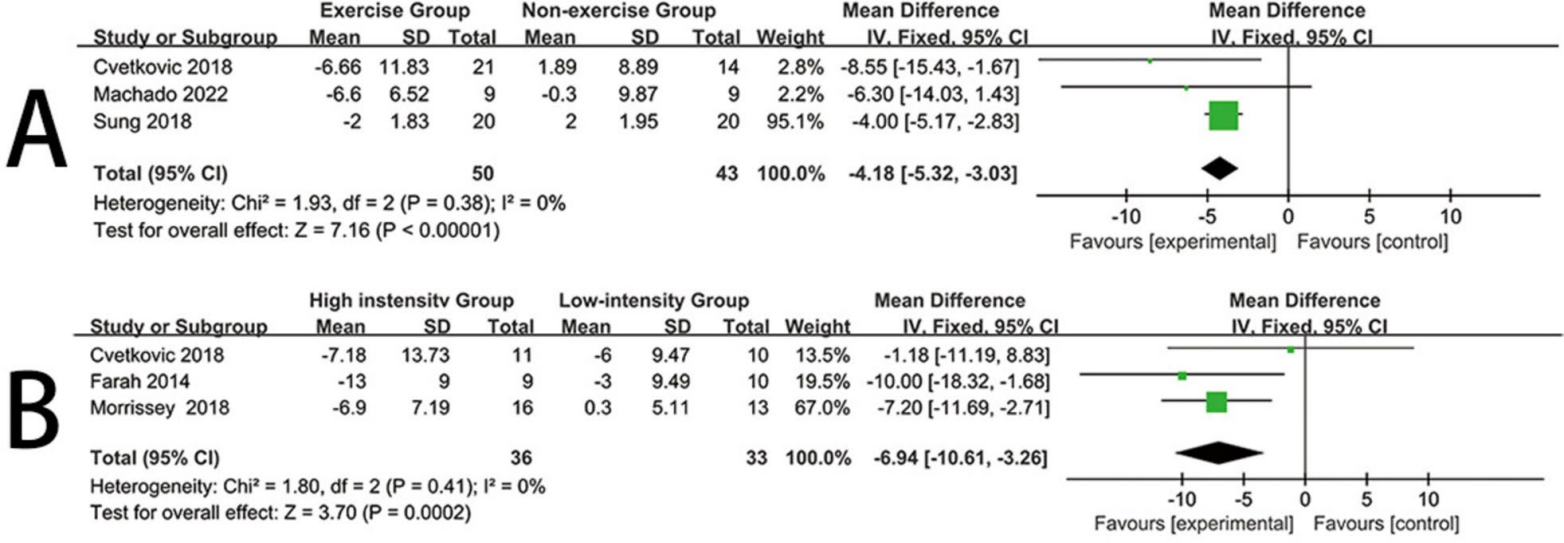
Forest plot for Diastolic blood pressure. **A** Forest plot of the exercise group vs the nonexercise group. **B** Forest plot of the high-instensity group vs the low-intensity group

Three trials [34, 36, 41] reported the impact of exercise interventions on diastolic blood pressure in obese children and adolescents, comprising 93 participants. Therefore, these three studies were included in the meta-analysis. Since there was no heterogeneity in this review (I^2^ = 0%, *P* = 0.38), a fixed-effects model was used. The results provided strong evidence that exercise interventions significantly reduced the diastolic blood pressure levels of obese children and adolescents compared to the control group (mean difference (MD) = -4.18, 95% confidence interval [CI] = [-5.32, -3.03], *P* < 0.00001) (Fig. 6A). Visual interpretation of funnel plots suggested no evidence of asymmetry (See Additional file 1).

Three trials [27, 36, 37] reported the impact of high-intensity and low-intensity exercise on diastolic blood pressure in obese children and adolescents, comprising 69 participants. Therefore, these three studies were included in the meta-analysis. Since there was no heterogeneity in this review (I^2^ = 0%, *P* = 0.41), a fixed-effects model was used. The results indicated that, when compared with low-intensity exercise, high-intensity exercise had a significantly greater effect on reducing diastolic blood pressure levels in obese children and adolescents (mean difference (MD) = -6.94, 95% confidence interval [CI] = [-10.61, -3.26], *P* = 0.0002) (Fig. 6B). Visual interpretation of funnel plots suggested no evidence of asymmetry (See Additional file 1).

## Discussion

**Fig. 7:**
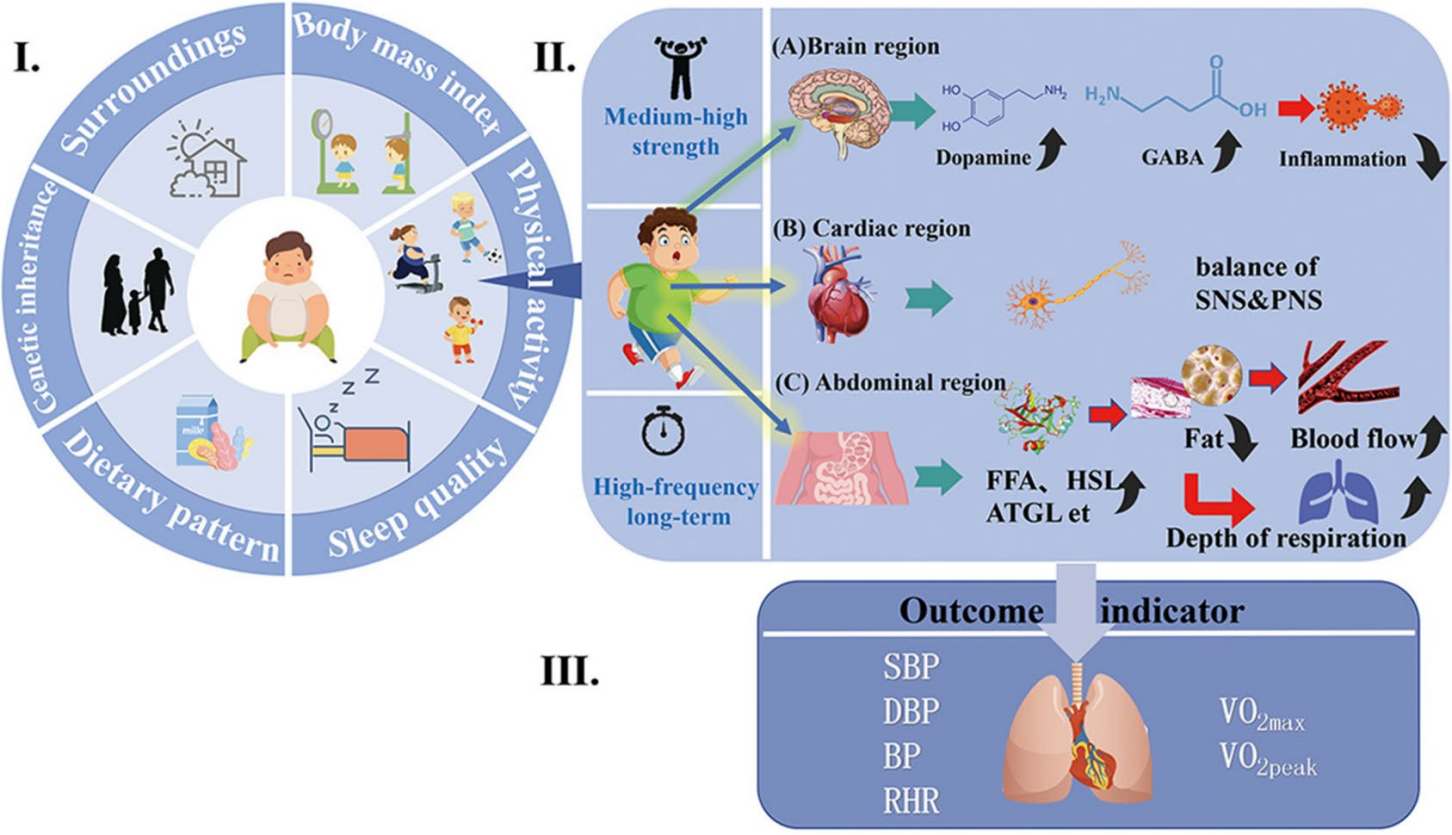
Summary of physical activity interventions for cardiopulmonary health in obese children and adolescents

CRF in obese children and adolescents is caused by different factors. The present discussion focuses on the effects of PA on CRF in obese children and adolescents. These study results indicate that physical activity can effectively reduce body weight, heart rate (HR), systolic blood pressure (SBP), and diastolic blood pressure (DBP) and increase VO_2max_ (maximal oxygen uptake) in obese children and adolescents. (Fig. 7I). Living environment, genetics, dietary patterns, sleep status, body mass index, and PA may be associated with CRF in obese children and adolescents, but not all influencing factors. II. The mechanism of PA intervention for CRF in obese children and adolescents mainly consists of three pathways that act on the (A) brain, (B) heart region, and (C) abdominal regions. When evaluating the effectiveness of different PA interventions for CRF in obese children and adolescents, the main focus is on the relationship between different exercise modes and intensities (Fig. 7).

### Mechanisms of physical activity interventions for CRF in obese children and adolescents

PA can be used to improve the cardiovascular health of obese children and adolescents. Heart rate variability (HRV) is thought to reflect the balance between sympathetic and parasympathetic mediators in the heart, with the ability of the autonomic nervous system to respond dynamically to environmental changes, which results in increased HRV and is often considered indicative of a healthy heart [43]. Farahb suggests that resting heart rate in obese adolescents is reduced by HIT and that this reduction is caused by increased parasympathetic modulation of the heart, as indicated by increases in mean interval RR, PNN50 and logHF after HIT. The aerobic exercise training program improved cardiovascular fitness in both the low- and high -intensity groups, but the greater increase in VO_2peak_ observed in the HIT group may be related to increased parasympathetic regulation [27] (Fig. 7II (A)).

PA reduces autoimmune inflammation in obese children and adolescents. PA increases the levels of neurotransmitters in the brain regions of obese children and adolescents, dopamine and the amino acid gamma-aminobutyric acid (GABA) in plasma and in different brain regions of humans [44–46]. Low CRF levels are associated with inflammation [47]. Deficiency of PA in childhood obesity leads to impairment of dopamine synthesis, release and receptor function (mainly in cells of the nervous system) [48]. Obesity may impair the dopaminergic and GABAergic systems [48]. Whereas dopaminergic and GABAergic neurons can be expressed in different types of immune cells and have different roles in the immune system, increasing GABAergic activity reduces autoimmune inflammation [49, 50]. The negative effects of obesity on the dopaminergic and GABAergic systems can be reduced through PA or exercise, thereby increasing CRF levels (Fig. 7II (B)).

PA improves fat metabolism in obese children and adolescents. Obese children and adolescents have higher levels of fat than the normal population, especially in the abdomen. As a result, obese children and adolescents have high serum levels of homocysteine compared to normal weight populations [51], leading to reduced vascular endothelial function, which in turn predisposes to many cardiovascular and cerebrovascular diseases [52]. PA can reduce fat in obese children and adolescents in several ways. First, PA increases the amount of free fatty acids (FFAs) in the blood of obese children and adolescents. The large amount of free fatty acids in the blood allows for adequate oxidation of fat and the breakdown of fat that has built up in the blood vessels. The vascular pathways become more elastic, enhancing the efficiency of oxygen delivery and improving the effectiveness of the circulatory system, achieving an improved cardiorespiratory system [53]. PA can increase the activity of a number of enzymes. Long-term aerobic training leads to enhanced lipid oxidase activity and upregulation of skeletal muscle lipid-droplet proteins (perilipins), hormone-sensitive lipase (HSL), and adipose triglyceride lipase (ATGL) levels [54]. Fat degradation is achieved by increasing the activity of the enzyme. Second, there is also extensive research confirming that the accumulation of fat in the central region of the body compresses the abdominal space, thereby causing the septum to rise reducing the depth of breathing and affecting one's cardiorespiratory function [53]. PA reduces abdominal fat accumulation, expands abdominal space and improves breathing depth, thereby improving cardiorespiratory function in obese children and adolescents. The mechanism of this process is the degradation of fat through a PA intervention, which results in improved cardiorespiratory function (Fig. 7II (C)).

### Effectiveness of different physical activity interventions for CRF in adolescents with obesity

There are differences in the effects of interventions for different types of PA.PA covered in the literature in this study included aerobic exercise (AE), augmented training (PLT), physical games (the MOVI-daFIT intervention), moderate-to-vigorous physical activity (MVPA), multidisciplinary weight loss programs (Kids4Fit), training at the maximal-fat-oxidation intensity (FAT_max_), and high intensity interval training (HIIT). School-based HIIT programmes can improve aerobic capacity in overweight and obese children. A study by Martínez et al. found that a 12-week extracurricular strength training programme improved CRF levels in children and adolescents more than a traditional low-intensity PA programme, mainly because high-intensity interval training improved VO_2peak_, VO_2max_, and EPOC [16]. In addition, Leeuwen et al. [10] used Kids4Fit as an intervention to enhance CRF, with two weekly interventions for the initial six weeks and one weekly intervention for the final six weeks. At the time of this study, significant positive effects on CRF were also noted in overweight and obese children, but after the intervention, CRF gradually decreased. Improvements in CRF in overweight and obese children are greatly affected when two additional exercises are added to the regular physical education programme, including exercises to improve coordination, strength, endurance, speed and flexibility [21]. Several studies have shown that high-intensity interval training (HIIT) is effective in improving CRF in obese children and adolescents [24, 55, 56].Martínez-Vizcaíno et al. studied mainly female children and adolescents and noted that HIIT improved girls' CRF during a school year [13]. In addition, scholars such as Espinoza-Silva have applied strategies of fitness equipment such as bicycles and treadmills as well as basic motor skills (running, jumping, throwing) in high-intensity programs [39]. The above literature demonstrates that HIIT is most effective in improving CRF in obese children and adolescents. Most forms of PA are based on treadmill walking or running, which has the advantage of being easy to monitor and organise; however, long-term treadmill interventions are limited in that they have no way to motivate and interest young obese children. Some studies have also involved sports games, which are more likely to attract the interest of obese children and adolescents, but the low intensity of exercise in sports games makes it difficult to achieve the goal of improving CRF. Therefore, it is important to target this group of obese children and adolescents both to stimulate their interest in PA and to achieve the goal of improving CRF. A combination of different sports within an intervention is recommended to avoid the boredom associated with a single activity and to complement each other activity in the programme (Fig. 7III).

There were also differences in the effects of exercise intensity on the intervention. The results showed that high-intensity exercise had a more significant effect on VO_2max_ and DBP in obese children and adolescents than low- to moderate-intensity exercise. First, involvement in mild-to-moderate PA may not be sufficient to improve CRF in children, and the effective dose of PA (i.e., PA intensity × PA duration) may be much higher in children than in adults [57]. Play sessions can be provided as a form of exercise at recess and before and after school. However, this is not sufficient to improve children's CRF, as it is not sufficient to achieve recommended 60 min/day of MPVA for children [37]. Second, the conclusions of the study by Sung et al. suggest that the amount and frequency of PA (preferably more than three times a week) is critical for improving CRF in children [34]. On the other hand, Martínez, S.R et al. demonstrated that the VO_2max_ of overweight children was increased in just three months while performing HIIT training twice a week as well as other physical activity, with the intervention effect becoming more pronounced when the intervention period was longer [16]. Kids4Fit is a good school-based intervention program for CRF in obese children, but daily interventions are necessary and longer intervention cycles are required for CRF to continue to improve. From the above literature, it appears that lower-intensity PA is not sufficient to improve CRF in obese children and adolescents, but a large body of literature demonstrates that moderate-to-high intensity PA has a significant effect on CRF in obese children and adolescents. High-frequency, long-term interventions have been shown to be more effective than short-term interventions (Fig. 7III).

### Other influencing factors

In terms of the individual, the chronological age (actual age), biological maturity, height and body mass of obese children and adolescents, have an impact on CRF [58]. Some studies have shown that the ratio of fruit, and vegetable intake to other nutritional intake of obese children and adolescents can have an impact on CRF [59]. It is possible that the quality of sleep and sedentary behaviour of obese children and adolescents may have some impact on CRF. Caloric restriction combined with exercise may lead to greater weight loss and better effects on metabolic and cardiovascular parameters [60]. There are also studies that combine factors such as sleep, diet and exercise to explore the effects of the combination of different factors on physical health indicators in children and adolescents. Although the population studied was not obese children and adolescents, and CRF has not been studied in depth, it is suggested to some extent that a combination of different factors may have an impact on CRF in obese children and adolescents.

On an objective level, first, environmental factors may influence CRF in obese children and adolescents. The environment and culture in which children and young people live varies from region to region, and environmental factors such as family upbringing and exercise philosophies may influence CRF. Living conditions, air pollution and other environmental factors in obese children and adolescents also contribute to asthma, thus affecting CRF. Second, genetics plays a crucial role in the physical health of children and adolescents, and good genetics, including parental health and education, will also improve CRF (Fig. 7I).

### Strengths, limitations and future prospects of this study

One of the strengths of this review is the strict adherence to scientific search procedures and evaluation methods. The second strength is the novelty of the perspective, which explores the mechanisms of PA intervention in CRF in obese children and adolescents from different regions of the body and the effects of the intervention from different perspectives of PA. Limitations of this review are as follows. First, we screened only two major databases and we deliberately decided to exclude grey literature, thus not necessarily including all relevant literature. The majority of the included literature used an RCT design, with a relatively homogenous type of experiment. Second, obese children and adolescents in different regions are influenced by different sociocultural and ethnic differences, and participants' PA levels and motivations were not clear. Third, it is not possible to determine whether there are other relevant factors affecting PA levels that influence the actual effect of CRF. Fourth, CRF assessment methods vary from study to study, which may lead to heterogeneity and bias in the overall effect estimates. In future studies, first, we propose a sex-differentiated comparison of CRF in adolescents with obesity, where physiological differences between boys and girls are likely to lead to different outcomes. Second, the age range of obese children and adolescents is 5–18 years old, and each stage of child and adolescent development has different physical and psychological characteristics. The delineation of age stages allows for relatively accurate problem solving. Again, future research needs to include improved and standardised the research methods, particularly in PA and CRF assessments.

## Conclusion

This systematic review mainly shows that PA can improve CRF in obese children and adolescents. The type of PA directly affects the interest in participation of obese children and adolescents, and the effects of different PA modes on CRF have not been explored in the literature. The PA intensity directly impacts the effect of the intervention, with the most significant effect being derived from moderate- to high-intensity PA, as well as high-frequency, long-term interventions, compared with short-term interventions. The three pathways to enhance CRF in obese children and adolescents are through PA, improving cardiovascular health, and reducing autoimmune inflammation and fat metabolism. The effects of the intervention are mainly reflected in maximum oxygen uptake, peak oxygen uptake, heart rate and resting heart rate, systolic blood pressure and diastolic blood pressure. Other factors that influence CRF in obese children and adolescents include genetic inheritance, living environment, dietary patterns and sleep.

### Supplementary Information

**Additional file 1: Supplementary Table S1.** Detailed search strategy in three databases. **Figure 1.** Funnel plot for Weight. (A) Funnel plot of the exercise group vs the nonexercise group. (B) Funnel plot of the high-instensity group vs the low-intensity group. **Figure 2.** Funnel plot for VO2max. (A) Funnel plot of the exercise group vs the nonexercise group. (B) Funnel plot of the high-instensity group vs the low-intensity group. **Figure 3.** Funnel plot for Heart rate. (A) Funnel plot of the exercise group vs the nonexercise group. (B) Funnel plot of the high-instensity group vs the low-intensity group. **Figure 4.** Funnel plot for Systolic blood pressure. (A) Funnel plot of the exercise group vs the nonexercise group. (B) Funnel plot of the high-instensity group vs the low-intensity group. **Figure 5.** Funnel plot for Systolic blood pressure. (A) Funnel plot of the exercise group vs the nonexercise group. (B) Funnel plot of the high-instensity group vs the low-intensity group.

## Data Availability

All data generated or analysed in this study are included in this published article [and its supplementary information file].
