# Supplementary material for: Physical activity interventions for cardiopulmonary fitness in obese children and adolescents: a systematic review and meta-analysis
Source: BMC Pediatr. 2023 Nov 6;23:558. doi: 10.1186/s12887-023-04381-8 (PMC10626758; doi:10.1186/s12887-023-04381-8)
Supplement: Supplementary file 1 — Additional file 1: Supplementary Table S1. Detailed search strategy in three databases. Figure 1. Funnel plot for Weight. (A) Funnel plot of the exercise group vs the nonexercise group. (B) Funnel plot of the high-instensity group vs the low-intensity group. Figure 2. Funnel plot for VO2max. (A) Funnel plot of the exercise group vs the nonexercise group. (B) Funnel plot of the high-instensity group vs the low-intensity group. Figure 3. Funnel plot for Heart rate. (A) Funnel plot of the exercise group vs the nonexercise group. (B) Funnel plot of the high-instensity group vs the low-intensity group. Figure 4. Funnel plot for Systolic blood pressure. (A) Funnel plot of the exercise group vs the nonexercise group. (B) Funnel plot of the high-instensity group vs the low-intensity group. Figure 5. Funnel plot for Systolic blood pressure. (A) Funnel plot of the exercise group vs the nonexercise group. (B) Funnel plot of the high-instensity group vs the low-intensity group. [file 12887_2023_4381_MOESM1_ESM.docx]

| Supplementary Table S1. Detailed search strategy in three databases. | |
| --- | --- |
| Database | Search strategy |
| Pubmed | (((("Exercise"[Mesh]) OR ((((((((((((((((((((((((Physical Activity) OR (Activities, Physical)) OR (Activity, Physical)) OR (Physical Activities)) OR (Exercise, Physical)) OR (Exercises, Physical)) OR (Physical Exercise)) OR (Physical Exercises)) OR (Acute Exercise)) OR (Acute Exercises)) OR (Exercise, Acute)) OR (Exercises, Acute)) OR (Exercise, Isometric)) OR (Exercises, Isometric)) OR (Isometric Exercises)) OR (Isometric Exercise)) OR (Exercise, Aerobic)) OR (Aerobic Exercise)) OR (Aerobic Exercises)) OR (Exercises, Aerobic)) OR (Exercise Training)) OR (Exercise Trainings)) OR (Training, Exercise)) OR (Trainings, Exercise))) AND ("Obesity"[Mesh])) AND (((((((((((((((((Adolescence) OR (Teens)) OR (Teen)) OR (Teenagers)) OR (Teenager)) OR (Youth)) OR (Youths)) OR (Adolescents, Female)) OR (Adolescent, Female)) OR (Female Adolescent)) OR (Female Adolescents)) OR (Adolescents, Male)) OR (Adolescent, Male)) OR (Male Adolescent)) OR (Male Adolescents)) OR ("Adolescent"[Mesh])) OR ((Children) OR ("Child"[Mesh])))) AND ((Fitness, Cardiorespiratory) OR ("Cardiorespiratory Fitness"[Mesh])) |
| Web of Science | 1 TS=(Exercises) OR TS=(Physical Activity) OR TS=(Activities, Physical) OR TS=(Activity, Physical) OR TS=(Physical Activities) OR TS=(Exercise, Physical) OR TS=(Exercises, Physical) OR TS=(Physical Exercise) OR TS=(Physical Exercises) OR TS=(Acute Exercise) OR TS=(Acute Exercises) OR TS=(Exercises, Acute) OR TS=(Exercise, Acute) OR TS=(Exercise, Isometric) OR TS=(Exercises, Isometric) OR TS=(Isometric Exercises) OR TS=(Isometric Exercise) OR TS=(Exercise, Aerobic) OR TS=(Aerobic Exercise) OR TS=(Aerobic Exercises) OR TS=(Exercises, Aerobic) OR TS=(Exercise Training) OR TS=(Exercise Trainings) OR TS=(Training, Exercise) OR TS=(Trainings, Exercise)  2 TS=(Adolescents) OR TS=(Adolescence) OR TS=(Teens) OR TS=(Teen) OR TS=(Teenagers) OR TS=(Teenager) OR TS=(Youth) OR TS=(Youths) OR TS=(Adolescents, Female) OR TS=(Adolescent, Female) OR TS=(Female Adolescent) OR TS=(Female Adolescents) OR TS=(Adolescents, Male) OR TS=(Adolescent, Male) OR TS=(Male Adolescent) OR TS=(Male Adolescents) OR TS=(Children) OR TS=(Child)  3 TS=(Obesity)  4 TS=(Cardiorespiratory Fitness) OR TS=(Fitness, Cardiorespiratory)  4 #1 AND #2 AND #3 AND #4 |
|  | |


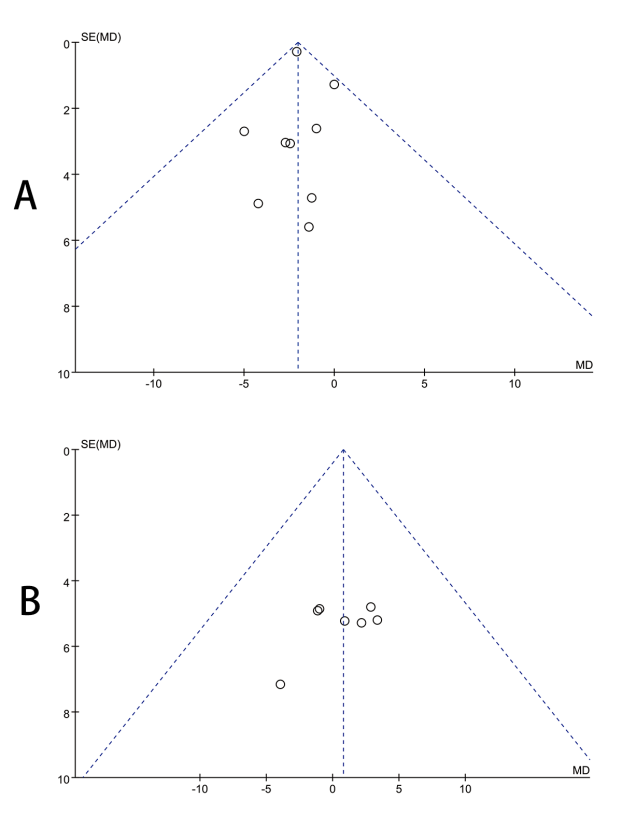


Figure 1 Funnel plot for Weight. (A) Funnel plot of the exercise group vs the nonexercise group. (B) Funnel plot of the high-instensity group vs the low-intensity group.


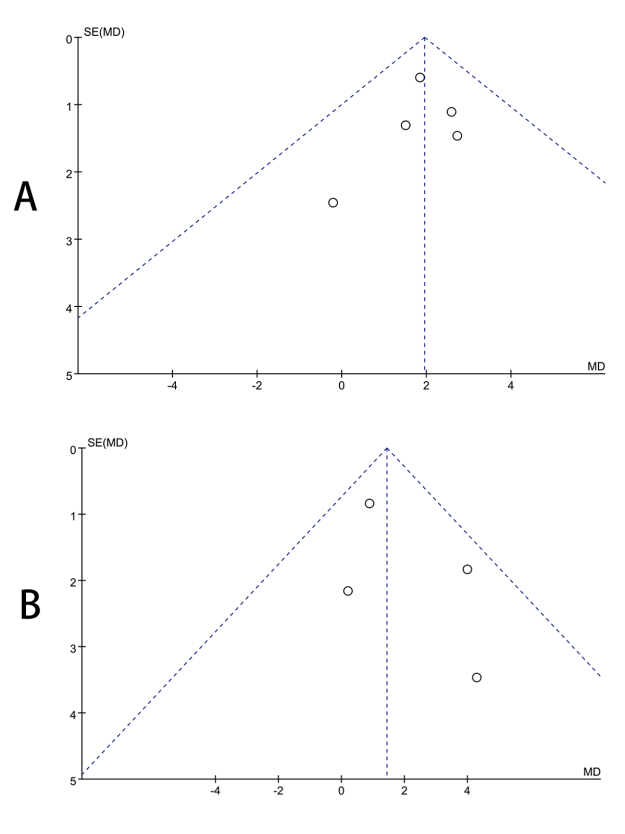


Figure 2 Funnel plot for VO_2max_. (A) Funnel plot of the exercise group vs the nonexercise group. (B) Funnel plot of the high-instensity group vs the low-intensity group.


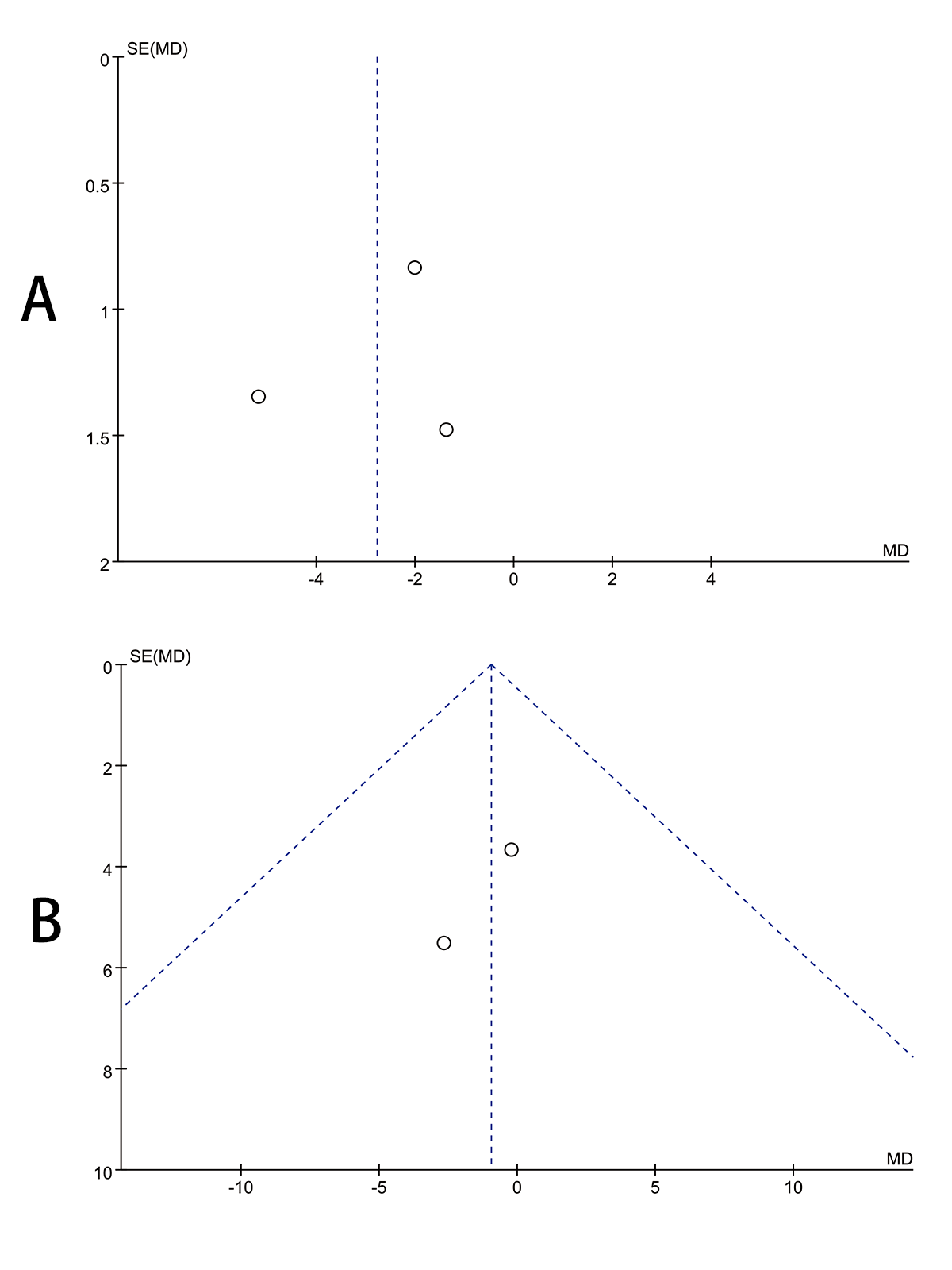


Figure 3 Funnel plot for Heart rate. (A) Funnel plot of the exercise group vs the nonexercise group. (B) Funnel plot of the high-instensity group vs the low-intensity group.


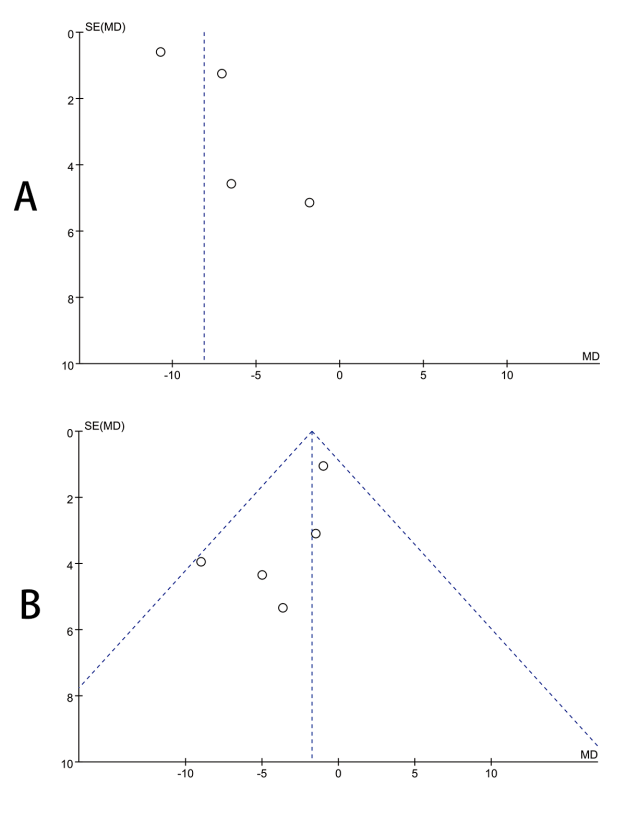


Figure 4 Funnel plot for Systolic blood pressure. (A) Funnel plot of the exercise group vs the nonexercise group. (B) Funnel plot of the high-instensity group vs the low-intensity group.


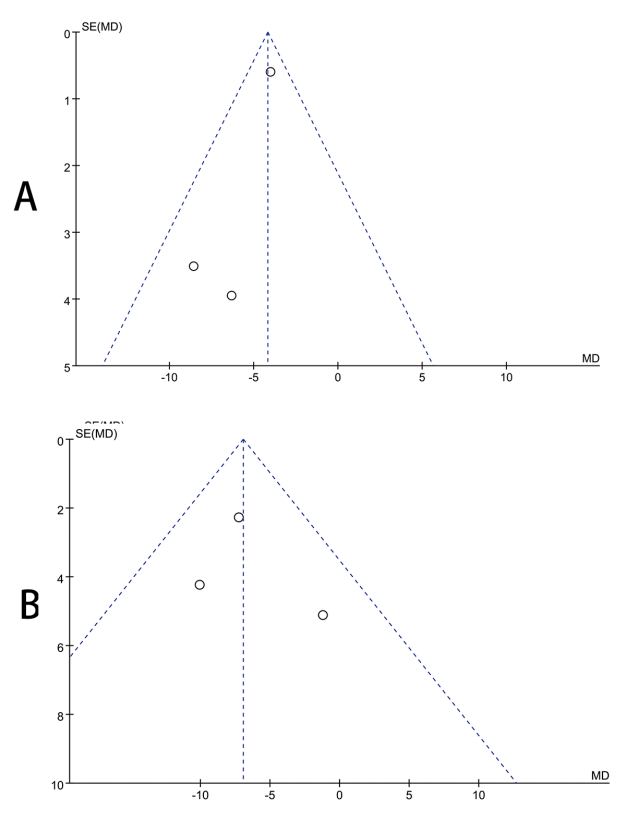


Figure 5 Funnel plot for Systolic blood pressure. (A) Funnel plot of the exercise group vs the nonexercise group. (B) Funnel plot of the high-instensity group vs the low-intensity group.
